# Mechanical Thrombectomy for Acute Stroke Due to Large-Vessel Occlusion Presenting With Mild Symptoms

**DOI:** 10.3389/fneur.2021.739267

**Published:** 2021-10-28

**Authors:** Feifeng Liu, Hao Shen, Chen Chen, Huan Bao, Lian Zuo, Xiahong Xu, Yumei Yang, Alexia Cochrane, Yaping Xiao, Gang Li

**Affiliations:** ^1^Department of Neurology, Shanghai East Hospital, School of Medicine, Tongji University, Shanghai, China; ^2^University of Edinburgh Medical School, Edinburgh, United Kingdom

**Keywords:** acute large vessel occlusion, thrombectomy, mild stroke, outcome, prognosis

## Abstract

**Purpose:** To evaluate the safety and efficacy of mechanical thrombectomy (MT) for acute stroke due to large vessel occlusion (LVO), presenting with mild symptoms.

**Methods:** A prospective cohort study of patients with mild ischemic stroke and LVO was conducted. Patients were divided into two groups: MT group or best medical management (MM) group. Propensity score matching (PSM) was conducted to reduce the confounding bias between the groups. The primary outcome was functional independence at 90 days. The safety outcome was symptomatic intracranial hemorrhage (sICH). Univariate and multivariate logistic regression analyses were used to identify the independent factors associated with outcomes.

**Results:** Among the 105 included patients, 43 were in the MT group and 62 in the MM group. Forty-three pairs of patients were generated after PSM. There were no significant differences in sICH rates between two groups (*p* = 1.000). The MT group had a higher proportion of independent outcomes (83.7% MT vs. 67.4% MM; OR 2.483; 95% CI 0.886–6.959; *p* = 0.079) and excellent outcomes (76.7% MT vs. 51.2% MM; OR 3.150; 95% CI 1.247–7.954; *p* = 0.013) compared to the MM group, especially in patients with stroke of the anterior circulation (*p* < 0.05). Multivariate logistic regression analysis showed that small infarct core volume (*p* = 0.015) and MT treatment (*p* = 0.013) were independently associated with excellent outcomes.

**Conclusions:** Our results suggest that MT in stroke patients, presenting with mild symptoms, due to acute LVO in the anterior circulation may be associated with satisfactory clinical outcomes.

**Clinical Trial Registration:** ClinicalTrials.gov, identifier: NCT04526756.

## Introduction

Mechanical thrombectomy (MT) has been globally acknowledged as a standard treatment for patients with acute ischemic stroke patients (AIS) and large vessel occlusion (LVO). Some randomized trials (1–7) provided level 1a evidence for MT in patients with LVO in the anterior circulation (internal carotid artery and middle cerebral artery M1) with a National Institutes of Health Stroke Scale (NIHSS) score of ≥6 (8, 9). However, mild stroke cannot be considered as a benign condition, with approximately one-third of patients becoming functionally dependent after 90 days (10–12). Large-scale prospective studies have shown that the NIHSS score is not a precise predictor of LVO in patients with AIS (13, 14), as nearly 10% patients with NIHSS score <6 suffered from LVO. Furthermore, patients with LVO presenting with a low NIHSS score were found to be a high risk of clinical worsening of the condition, leading to poor outcome (15). It is still unclear whether patients with minor stroke and LVO can benefit from MT. Several studies have focused on this topic, but the results are inconsistent (16–19). The aim of this prospective study is to evaluate the relationship between MT and the clinical outcome of acute LVO with mild symptoms.

## Materials and Methods

### Study Design

We conducted a prospective cohort study on patients with LVO and mild symptoms from a comprehensive stroke center in Shanghai, China, between August 1, 2016, and March 31, 2020. The inclusion criteria were as follows: (1) time from onset or last seen well of the symptoms ≤24 h; (2) age ≥18 years; (3) NIHSS score < 6; (4) LVO including middle cerebral artery M1, proximal M2 segment, intracranial internal carotid artery, basilar artery, and posterior cerebral artery P1 occlusion detected by computed tomography (CT) angiography (CTA); and (5) anterior circulation LVO with infarct core volume ≤50 ml and mismatch ratio >1.8. Patients with prior disability [the modified Rankin Scale (mRS) score of >2] and occlusive artery spontaneous recanalization before thrombectomy and those with neurological worsening in the medical management (MM) group with rescue MT were excluded.

The study was approved by the institutional ethics committee of Shanghai East Hospital, and a written informed consent was obtained from each participant.

### Measurements

All patients underwent an initial imaging protocol including non-enhanced CT scan. When patients with mild stroke (NIHSS score <6) are considered as anterior circulation stroke presenting with cortical symptoms (such as mild aphasia, somnolence, slow-mindedness) or posterior circulation stroke presenting with continuous vertigo and bilateral pathological signs within 24 h, they were suspected with LVO and received the emergency whole-brain CT perfusion (CTP), which can obtain CTA and CTP images at the same time to judge whether there is LVO and penumbra. If they met the inclusion criteria mentioned above, patients were informed of the advantages and disadvantages of the MT and MM treatment and decided which treatment to receive. The study subjects were divided into two groups: MT group, and best MM group. The following data were collected: (1) demographics; (2) time of onset, admission, thrombolysis, CTP examination, puncture, and reperfusion; (3) medical history; and (4) NIHSS score before evaluation of MT.

Computed tomography perfusion/Computed tomography angiography images were acquired using a 320-slice CT scanner (Aquilion ONE, Toshiba Medical Systems, Otawara, Japan). Computed tomography perfusion data were analyzed using a commercial software (MiStar; Apollo Medical Imaging Technology, Melbourne, Australia). Hypoperfusion volume and infarct core volume were calculated using previously validated thresholds [hypoperfused lesion: delay time (DT) >3 s, infarct core: relative cerebral blood flow (rCBF) <30%, severe hypoperfused lesion: DT >6 s] (20–22). The mismatch ratio was defined as the proportion of hypoperfusion volume to the infarct core volume (22). Acute cerebral collateral flow was quantified using the volume ratio of severely delayed contrast transit tissue (DT >6 s within the DT >3 s perfusion lesion) (23).

Solitaire stent placement and manual aspiration were performed as first-line endovascular treatment. When stent retriever thrombectomy failed, an intra-arterial recombinant tissue plasminogen activator (rtPA) was administered or a stent was placed. Successful revascularization was defined as a modified thrombolysis in cerebral infarction (TICI) grade 2b or 3 (24).

The mRS was assessed at 3 months *via* telephone by a trained staff who was unaware of the patients' treatment group. The mRS score was recorded as 6 at the 3-month follow-up when the patients died in the hospital. The primary outcome was defined as functional independence (mRS score of 0–2) at 90 days. We also evaluated excellent clinical outcomes (mRS score of 0–1) at 90 days, NIHSS score (a NIHSS score of 42 was assigned in case of death) and mRS score at 7 days, symptomatic intracranial hemorrhage (sICH), in-hospital mortality, and at 90 days as secondary outcomes. Symptomatic intracranial hemorrhage was defined according to the European Cooperative Acute Stroke Study (ECASS)-III criteria (25).

### Statistical Analysis

Statistical analysis for categorical variables included the Chi-square test and Fisher's exact test when cell sizes were small. Quantitative data were described as mean ± standard deviation (SD) if normally distributed or the median, i.e., interquartile range (IQR), and analyzed using Student's *t*-test or the Mann–Whitney test. Logistic regression analysis was performed to identify the independent predictors of clinical outcomes. Variables with a *p*-value of < 0.15 in the univariate analysis of the clinical outcome were included in the multivariate logistic regression. The Matchit package in R language was used to conduct the propensity score matching (PSM) analysis (nearest neighbor 1:1 matching). Other statistical analyses were conducted using SPSS version 22.0, for Windows (SPSS Inc., Chicago, IL, USA). Statistical significance was set at *p* < 0.05.

## Results

**Figure 1:**
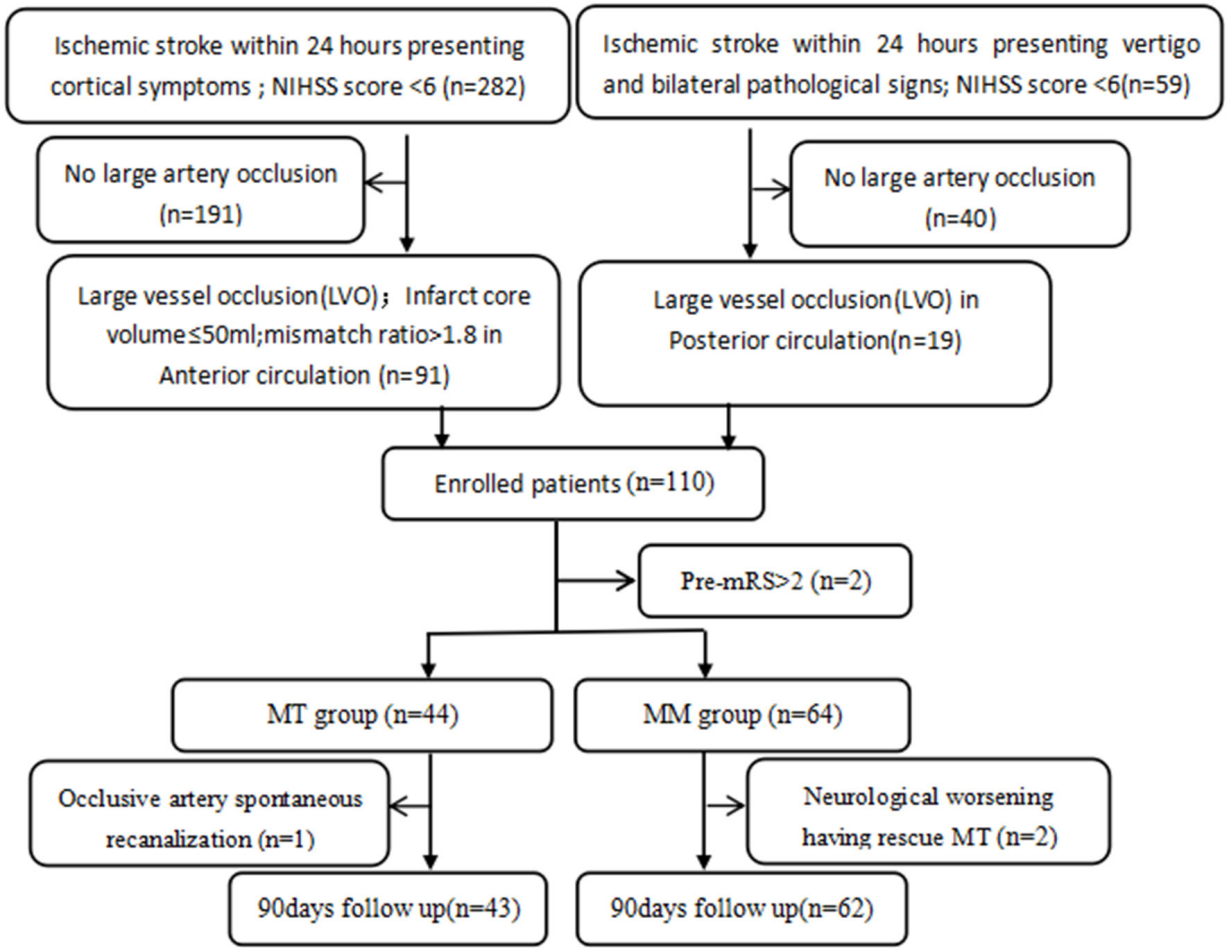
The study flowchart. NIHSS, National Institutes of Health Stroke Scale; mRS, modified Rankin Scale; MT, mechanical thrombectomy; MM, medical management; LVO including middle cerebral artery M1, proximal M2 segment, intracranial internal carotid artery, basilar artery, and posterior cerebral artery P1 occlusion.

We initially identified 110 patients for this study. Of them, two patients were excluded because of a pre-stroke mRS >2, one in the MT group was excluded for spontaneous recanalization, and two patients in the MM group were excluded for rescue MT due to neurological worsening. Of the two excluded patients, one was excluded because he had acute occlusion of the contralateral middle cerebral artery during hospitalization, which was not related to this stroke. Another one was treated by interventional surgery due to the aggravation of cerebral infarction symptoms. We excluded this patient because we considered that there may be a correlation between thrombectomy and the prognosis. Thus, finally a total of 105 patients (64.8% male) with a mean age of 68.8 years (SD 11.7) and median NIHSS score of 5 (4,5) were included. Forty-three patients (41%) were placed in the MT group, and 62 (59%) patients were in the MM group. All 105 patients were followed up at 90 days. The summary of the study design is presented as a flowchart in Figure 1. Forty-three pairs of patients were generated after PSM.

Baseline demographics, medical history, and perfusion analyses results were comparable between the two groups. Before PSM, the MT group had more MCA-M1 (46.5 vs. 38.7%), less MCA-M2 (7 vs. 25.8%), and less posterior circulation occlusions (9.3 vs. 22.6%) than the MM group (*p* = 0.004). Moreover, these differences were reduced after PSM (Table 1).

**Table 1:** Comparisons of baseline clinical profiles and CTP data between thrombectomy and medical management groups before and after PSM.

**Characteristic**		**Before PSM**			**After PSM**		
		**MT group (*n* = 43)**	**MT group (*n* = 62)**	***p*-value**	**MT group (*n* = 43)**	**MM group (*n* = 43)**	***p*-value**
**Baseline demographic and clinical data**							
**Age**, years, mean ± SD		66.51 ± 9.26	67.02 ± 13.18	0.829	66.51 ± 9.26	66.86 ± 12.65	0.884
**Sex**, male, *n* (%)		27 (62.8)	41 (66.1)	0.725	27 (62.8)	27 (62.8)	1.000
**Medical history**, *n* (%)							
Hypertension		27 (62.8)	45 (72.6)	0.288	27 (62.8)	29 (67.4)	0.651
Diabetes mellitus		8 (18.6)	14 (22.6)	0.623	8 (18.6)	10 (23.3)	0.596
Dyslipidemia		6 (14.0)	10 (16.1)	0.760	6 (14.0)	6 (14.0)	1.000
Ischemic stroke		7 (16.3)	8 (12.9)	0.627	7 (16.3)	7 (16.3)	1.000
Cardioembolic source		16 (37.2)	20 (32.3)	0.599	16 (37.2)	15 (34.9)	0.822
Smoking		15 (34.9)	23 (37.1)	0.816	15 (34.9)	14 (32.6)	0.820
Taking antiplatelet prior to stroke		8 (18.6)	12 (19.4)	0.923	8 (18.6)	8 (18.6)	1.000
Taking anticoagulant prior to stroke		3 (7.0)	3 (4.8)	0.687	3 (7.0)	3 (7.0)	1.000
**Glucose at admission**, mmol/L, median (IQR)		6.7 (5.9, 7.9)	6.95 (6.1, 8.9)	0.444	6.7 (5.9, 7.9)	6.6 (5.9, 8.9)	0.782
**Intravenous rtPA**, *n* (%)		19 (44.2)	26 (41.9)	0.819	19 (44.2)	20 (46.5)	0.829
**DNT**, min, median (IQR)		34 (28, 45)	34 (27, 48)	0.827	34 (28, 45)	32 (24.5, 46.5)	0.844
**NIHSS before evaluation of thrombectomy**, median (IQR)		5 (4, 5)	5 (3, 5)	0.138	5 (4, 5)	5 (4, 5)	0.835
**Onset to door time**, min, median (IQR)		121 (60, 309)	143 (60, 303)	0.604	121 (60, 309)	115 (59, 285)	0.900
**Onset to CTP time**, min, median (IQR)		219 (130, 415)	235 (151, 381)	0.555	219 (130, 415)	236 (148, 358)	0.832
**Onset to puncture time**, min, median (IQR)		360 (275, 543)	NA	NA	360 (275, 543)	NA	NA
**Onset to clot first reperfusion time**, min, median (IQR)		433 (345, 598)	NA	NA	433 (345, 598)	NA	NA
**Local anesthesia**, *n* (%)		20 (46.5)	NA	NA	20 (46.5)	NA	NA
**Successful recanalization** (TICI 2b/3), *n* (%)		42 (97.7)	NA	NA	42 (97.7)	NA	NA
**Occlusion site**	MCA M1, *n* (%)	20 (46.5)	24 (38.7)	0.004	20 (46.5)	18 (41.9)	0.001
	MCA M2, *n* (%)	3 (7.0)	16 (25.8)		3 (7.0)	10 (23.3)	
	Proximal ICA, *n* (%)	5 (11.6)	4 (6.5)		5 (11.6)	3 (7.0)	
	Terminal ICA or tandem occlusion, *n* (%)	11 (25.6)	4 (6.5)		11 (25.6)	2 (4.7)	
	BA or PCA P1, *n* (%)	4 (9.3)	14 (22.6)		4 (9.3)	10 (23.3)	
**Baseline perfusion imaging data**							
**Infarct core**, ml, median (IQR)		6 (3, 25)	6.5 (3, 18)	0.747	6 (3, 25)	7 (3, 18)	0.694
**Penumbra**, ml, median (IQR)		89 (32, 118)	63 (23, 103)	0.062	89 (32, 118)	73 (24, 103)	0.240
**DT** **>** **3 s**, ml, median (IQR)		95 (37, 148)	75.5 (27, 123.3)	0.121	95 (37, 148)	95 (29, 125)	0.344
**DT** **>** **6 s**, ml, median (IQR)		19.0 (4.6, 36.8)	11.5 (3.9, 31.9)	0.265	19.0 (4.6, 36.8)	11 (5, 35)	0.426
**Mismatch ratio**, median (IQR)		10.5 (4.6, 18.5)	7.8 (3.0, 16.9)	0.171	10.5 (4.6, 18.5)	8.7 (3.5, 18.6)	0.392
**DT6/DT3 ratio**, median (IQR)		0.22 (0.11, 0.33)	0.18 (0.08, 0.30)	0.542	0.22 (0.11, 0.33)	0.18 (0.05, 0.30)	0.402

*Data are n (%), mean (SD), or median (IQR). p-values are based on chi-square, T-test, or Wilcoxon signed-rank test. PSM, propensity score matching; MT, mechanical thrombectomy; MM, medical management; rtPA, recombinant tissue plasminogen activator; DNT, door to needle time; NA, not applicable; IQR, interquartile range; NIHSS, National Institutes of Health Stroke Scale; DT, delay time; ICA, internal carotid artery; MCA, middle cerebral artery; PCA, posterior cerebral artery; BA, basilar artery*.

With respect to clinical outcomes, Before PSM, patients in the MT group had a significantly higher proportion of independence (83.7 vs. 62.9%; OR 3.033; 95% CI 1.162–7.919; *p* = 0.02) and excellent clinical outcomes (76.7% MT vs. 45.2% MM; OR 4.007; 95% CI 1.685–9.531; *p* = 0.001) than the MM group at 90 days, especially in the anterior circulation group. Patients treated with MT also had lower NIHSS and mRS scores at 7 days than those in the MM group (*p* < 0.05). Symptomatic intracranial hemorrhage rates (7% MT vs. 4.8% MM group; *p* = 0.687), in-hospital mortality (9.3% MT vs. 4.7% MM group; *p* = 0.714), incidence of pulmonary infection (23.3% MT vs. 22.6% MM group; *p* = 0.935), and length of hospitalization (12 days vs. 11 days, *p* = 0.131) were comparable between the groups (Table 2). In the univariate analysis, female, pre-mRS = 0, small infarct core volume (≤15 ml), and MT were identified as predictors of good outcomes. Multivariate logistic regression showed that a small infarct core volume (OR 3.275, 95% CI 1.221–8.783, *p* = 0.018; OR 3.102, 95% CI 1.156–8.323, *p* = 0.025) and MT (OR 3.320, 95% CI 1.163–9.479, *p* = 0.025; OR 4.442, 95% CI 1.737–11.359, *p* = 0.002) were associated with independence and excellent clinical outcome at 90 days (Table 3).

**Table 2:** Clinical outcomes of patients between thrombectomy and medical management group before and after PSM.

**Characteristic**	**Before PSM**				**After PSM**			
	**MT group (*n* = 43)**	**MT group (*n* = 62)**	***p*-value**	**OR (95% CI)**	**MT group (*n* = 43)**	**MM group (*n* = 43)**	***p*-value**	**OR (95% CI)**
**sICH**, ***n*** **(%)**	3 (7.0)	3 (4.8)	0.687	1.475 (0.283–7.679)	3 (7.0)	2 (4.7)	1.000	1.538 (0.244–9.70)
**Death in hospital**, ***n*** **(%)**	4 (9.3)	4 (4.7)	0.714	1.478 (0.351–6.303)	4 (9.3)	3 (7.0)	1.000	1.368 (0.287–6.512)
**Death at 90 days**, ***n*** **(%)**	5 (11.6)	4 (6.5)	0.482	1.908 (0.481–7.561)	5 (11.6)	3 (7.0)	0.713	1.754 (0.392–7.852)
**Pulmonary infection**, ***n*** **(%)**	10 (23.3)	14 (22.6)	0.935	1.039 (0.412–2.619)	10 (23.3)	10 (23.3)	1.000	1.000 (0.368–2.720)
**NIHSS score at 7 days**, median (IQR)	2 (1.5)	4 (2.75.7.25)	0.042		2 (1.5)	4 (2.8)	0.058	
**mRS at 7 days**, median (IQR)	1 (1.3)	3 (1.4)	0.033		1 (1.3)	2 (1.4)	0.078	
**mRS (0–1) at 90 days**, ***n*** **(%)**	33 (76.7)	28 (45.2)	0.001	4.007 (1.685–9.531)	33 (76.7)	22 (51.2)	0.013	3.15 (1.247–7.954)
Anterior circulation	31 (79.5)	20 (41.7)	0.000	5.425 (2.065–14.255)	31 (79.5)	16 (48.5)	0.006	4.117 (1.463–11.584)
Posterior circulation	2 (50.0%)	8 (57.1)	1.000	0.750 (0.081–6.958)	2 (50.0)	6 (60)	1.000	0.667 (0.065–6.871)
**mRS (0–2) at 90 days**, ***n*** **(%)**	36 (83.7)	39 (62.9)	0.002	3.033 (1.162–7.919)	36 (83.7)	29 (67.4)	0.079	2.483 (0.886–6.959)
Anterior circulation	34 (87.2)	29 (60.4)	0.005	4.455 (1.479–13.420)	34 (87.2)	22 (66.7)	0.037	3.40 (1.039–11.124)
Posterior circulation	2 (50.0)	10 (71.4)	0.569	0.400 (0.041–3.900)	2 (50.0)	7 (70)	0.580	0.429 (0.04–4.637)
**mRS at 90 days**, median (IQR)	1 (0.1)	2 (0.3)	0.039		1 (0.1)	1 (0.3)	0.096	
**Hospitalization days, day**, median (IQR)	12 (11.15)	11 (9.15)	0.131		12 (11.15)	11 (8.15)	0.120	

*Data are n (%), mean (SD), or median (IQR). P-values are based on chi-square, T test, or Wilcoxon signed-rank test; PSM, propensity score matching; MT, mechanical thrombectomy; MM, medical management; sICH, symptomatic intracranial hemorrhages; OR, odds ratio; CI, confidence interval; NIHSS, National Institute of Health Stroke Scale; mRS, modified Rankin Scale; IQR, interquartile range*.

**Table 3:** Univariate and multivariate analysis of determinants of good outcome before and after PSM.

	**Before PSM**				**After PSM**			
	**mRS (0–1) at 90 days**				**mRS (0–1) at 90 days**			
**Variables**	**Unadjusted OR (95% CI)**	***p*-value**	**Adjusted OR (95% CI)**	***p*-value**	**Unadjusted OR (95% CI)**	***p*-value**	**Adjusted OR (95% CI)**	***p*-value**
**Thrombectomy**	4.01 (1.69–9.53)	0.001	4.44 (1.74–11.36)	0.002	3.15 (1.25–7.95)	0.013	3.61 (1.31–9.93)	0.013
**Infarct core** **≤15 ml**	3.46 (1.43–8.36)	0.005	3.10 (1.16–8.32)	0.025	4.12 (1.54–11.50)	0.004	3.91 (1.30–11.74)	0.015
**Gender, male**	0.37 (0.16–0.88)	0.023	0.40 (0.16–1.04)	0.059	0.45 (0.17–1.18)	0.100	0.52 (0.18–1.49)	0.222
**Pre-mRS** **=** **1**	0.17 (0.02–1.55)	0.159	0.35 (0.03–3.71)	0.038	0.17 (0.02–1.74)	0.131	0.37 (0.03–4.16)	0.417
	**mRS (0–2) at 90 days**				**mRS (0–2) at 90 days**			
**Variables**	**Unadjusted OR (95% CI)**	***p*** **-value**	**Adjusted OR (95% CI)**	***p*** **-value**	**Unadjusted OR (95% CI)**	***p*** **-value**	**Adjusted OR (95% CI)**	***p*** **-value**
**Thrombectomy**	3.03 (1.16–7.92)	0.020	3.32 (1.16–9.48)	0.025	2.48 (0.89–6.96)	0.079	2.95 (0.92–9.50)	0.070
**Infarct core** **≤15 ml**	4.00 (1.61–9.96)	0.002	3.27 (1.22–8.88)	0.018	6.55 (2.22–19.28)	0.000	5.78 (1.81–18.42)	0.003
**Gender, male**	0.46 (0.17–1.20)	0.106	0.58 (0.20–1.63)	0.299	0.44 (0.14–1.35)	0.144	0.59 (0.17–2.04)	0.405
**Pre-mRS** **=** **1**	0.09 (0.01–0.82)	0.023	0.16 (0.02–1.66)	0.124	0.09 (0.01–0.96)	0.044	0.20 (0.02–2.30)	0.195

*Adjusted for sex, Pre-mRS, thrombectomy and infarct core **≤**15 ml. PSM, propensity score matching; mRS, modified Rankin Scale; OR, odds ratio; CI, confidence interval*.

Similar results were obtained after PSM analysis. Patients in the anterior circulation stroke, MT group, had a significantly higher proportion of independence (87.2 vs. 66.7%; *p* = 0.037) and excellent clinical outcomes (79.5% MT vs. 48.5% MM; *p* = 0.006) than the MM group at 90 days (Table 2). Multivariate logistic regression showed that a small infarct core volume (OR 3.91, 95% CI 1.299–11.744, *p* = 0.015) and MT (OR 3.612, 95% CI 1.314–9.927, *p* = 0.013) were associated with excellent clinical outcome at 90 days (Table 3).

## Discussion

This prospective cohort study of patients with mild ischemic stroke (NIHSS score <6) and LVO showed that MT treatment was independently associated with good functional outcome at 90 days, especially in anterior circulation post-procedure. Similar rates of sICH, in-hospital mortality, and pulmonary infection between groups may contribute to concerns regarding the safety of MT.

It is still unclear whether patients with acute mild ischemic stroke due to LVO can benefit from thrombectomy. To the best of our knowledge, there have been no randomized controlled trials on this topic, and observational studies have shown conflicting results. Our findings supported the results of the retrospective study by Haussen et al. (19); compared to medical therapy, thrombectomy in patients with LVO with mild symptoms (NIHSS 0–5) was associated with improved clinical outcomes. A multicenter-matched analysis (18) with a larger sample size also showed similar results. In addition, a meta-analysis (26) conducted in 2018 demonstrated results in favor of thrombectomy in patients with LVO stroke with minor or mild symptoms (NIHSS ≤ 8). However, the patients in the thrombectomy group were considerably younger, which may have been a major confounder. In contrast, ETIS registry investigators (27) reported similar proportions of favorable functional outcomes at 3 months between urgent MT and medical treatment with possible delayed MT matched with propensity score, but with nearly 20% management crossover. Similar results were reported in another multicenter cohort study (17); however, there was a signal toward benefit from MT in M1 occlusions (*p* = 0.07) (17). A recent meta-analysis (16) demonstrated that there was no difference in clinical outcomes in patients with LVO and NIHSS score <6 treated with MT or best MM. Our research adds to the knowledge in this field with regard to the eastern Chinese population.

In terms of safety, we found no significant difference in the rate of sICH and mortality between the MT and MM groups, which is consistent with the findings of some matched cohort studies (18, 27). Goyal et al. in a meta-analysis (16) found that MT was associated with higher rates of asymptomatic ICH (OR, 11.07; 95% CI, 1.31–93.53; *p* = 0.03), but with a similar rate of sICH between the two groups. Sarraj et al. (17) observed that sICH rates were higher in the MT group and were associated with higher mortality. Multiple passes of MT might be related to an increased risk of sICH. Patients in this study were included from nearly 10 years ago, and the development of thrombectomy technique may be associated with a decreased rate of complications and mortality. Overall, sICH and other complications are still major concerns for MT in patients with mild stroke.

There are several possible reasons behind the positive results in this study. First, the higher recanalization rate in this study (up to 97.7%) was comparable to that in a meta-analysis by Goyal et al. (84.5%) (16) and that reported by Sarraj et al. (78%) (17). A higher recanalization rate might be associated with a better outcome in the MT group, which was also supported by a retrospective analysis of the BEYOND-SWIFT registry study (28). Better outcomes were observed in patients with successful reperfusion. Second, perfusion imaging was used to select patients. In China, there were no clear recommendations on the guidelines for thrombectomy in patients with LVO and mild stroke (29, 30). No recommendation was described in the 2015 guideline, and MT can be considered after careful analysis and screening in the 2018 guideline. Even though there were no common rules to make a treatment decision for such condition, patients with smaller infarct cores and larger penumbra might prefer thrombectomy. In addition, treatment decision might also be affected by insurance status and family culture. Even though we used multivariate logistic regression and PSM to adjust for these confounders, our results might still be affected by indication bias.

### Strengths and Limitations

Our study was prospectively designed to explore the relationship between MT treatment in patients with mild stroke and associated functional outcomes. The baseline characteristics were almost balanced between groups providing ideally comparable populations. There were several limitations to this study, including the non-randomized study design with a limited sample size, which might have caused potential bias. The baseline NIHSS score in the MT group seemed higher than that in the MM group, and there were more patients with anterior circulation occlusion in the MT group. To overcome these drawbacks, PSM analysis was conducted to reduce the confounding bias. Also, we tried to adjust these imbalances with multivariate analysis. Furthermore, the study included both anterior and posterior circulation patients, which may reduce the credibility of the results. So we conducted a stratified analysis, suggesting that the benefit of thrombectomy is mainly reflected in the anterior circulation and there is no statistical difference between the two groups in the posterior circulation. Moreover, the power of the NIHSS score in evaluating the severity of posterior circulation and the reliability of perfusion imaging in the evaluation of posterior circulation is low, which may also have caused potential bias. Finally, not all patients presented with NIHSS <6 underwent emergency multimodal imaging to screen for LVO; only when stroke doctors considered that LVO cannot be completely excluded according to the patient's clinical symptoms would they give the patient multimodal imaging examination, which may lead to selection bias. Therefore, it is necessary to conduct a large multicenter randomized controlled trial comparing the MT with the best MM in patients with LVO presenting with minor symptoms.

## Conclusions

This study suggested that MT in patients presenting with mild stroke symptoms and acute LVO may be related to good clinical outcomes, especially in the anterior circulation, with a similar risk of sICH. This observational study adds evidence in support of thrombectomy in patients with mild stroke patients in the eastern Chinese population.

## Data Availability Statement

The original contributions presented in the study are included in the article/supplementary material, further inquiries can be directed to the corresponding author/s.

## Ethics Statement

The studies involving human participants were reviewed and approved by Ethics Committee of the Shanghai East Hospital. The patients/participants provided their written informed consent to participate in this study.

## Author Contributions

FL and HS conceived of and designed the study, wrote the first draft of the manuscript, and analyzed the data. GL designed the study and finalized the manuscript. All authors revised and approved the final manuscript.

## Funding

This work was supported by grants obtained from the Chinese Cardiovascular Association-V.G. fund (Grant No. 2017-CCA-VG-025), the Shanghai Key Clinical Discipline (Grant No. shslczdzk06103), the Outstanding Leaders Training Program of Pudong New Area Health System, Shanghai, China (Grant No. PWRl 2018-01), the Top-level Clinical Discipline Project of Shanghai Pudong (Grant No. PWYgf 2018-05), the Scientific Research Projects of Shanghai Health and Family Planning Commission (Grant No. 201640388), and the Registration Trial of the Microport Neurotech Stentriever for the Treatment of Acute Ischemic Stroke (Grant No. KPX2019-S12).

## Conflict of Interest

The authors declare that the research was conducted in the absence of any commercial or financial relationships that could be construed as a potential conflict of interest.

## Publisher's Note

All claims expressed in this article are solely those of the authors and do not necessarily represent those of their affiliated organizations, or those of the publisher, the editors and the reviewers. Any product that may be evaluated in this article, or claim that may be made by its manufacturer, is not guaranteed or endorsed by the publisher.

## Abbreviations

AIS: acute ischemic stroke
CT: computed tomography
CI: confidence interval
CTA: CT angiography
CTP: CT perfusion
DT: delay time
ECASS: European Cooperative Acute Stroke Study
IQR: interquartile range
LVO: large vessel occlusion
MT: mechanical thrombectomy
MM: medical management
mRS: modified Rankin scale
NIHSS: National Institutes of Health Stroke Scale
OR: odds ratio
PSM: propensity score matching
rtPA: recombinant tissue plasminogen activator
rCBF: relative cerebral blood flow
SD: standard deviation
sICH: symptomatic intracranial hemorrhage
TICI: thrombolysis in cerebral infarction.
